# INTRODUCING “X-VERSITY” THEORY: EXPERIENTIAL DIVERSITY IN DAILY LIFE AND ITS IMPLICATIONS FOR HEALTHY AGING

**DOI:** 10.1093/geroni/igae098.0344

**Published:** 2024-12-31

**Authors:** Rachel Koffer, Soomi Lee, Johanna Drewelies

**Affiliations:** Arizona State University, Phoenix, Arizona, United States; The Pennsylvania State University, University Park, Pennsylvania, United States; Max Planck Institute for Human Development, Berlin, Berlin, Germany

## Abstract

Lifespan developmental theories highlight that aging is characterized by gains and losses across different domains (e.g., social, emotional, physical, cognitive). Such gains and losses translate into aging-related change in experiences across various facets of daily life. However, much of present theory and empirical evidence has focused on overall quantity of experiences (e.g., total number of social activities) rather than how such experiences are spread across different types (e.g., whether a person spends relatively more of their time in one social activity compared to other activities). This presentation introduces the X-versity theory, in which we propose that experiential diversity (X-versity), conceptualized as rich and balanced experiences, is an important component of healthy and resilient aging. We present a conceptual framework that demonstrates the theoretical underpinnings associating person-environment interactions with differences in X-versity, and links X-versity with aging-related outcomes. Empirical evidence representing scientific advances in this field is then presented, particularly within the domains of activity, stressor, and emotion diversity. The empirical evidence will be used to demonstrate associations within the conceptual framework, and how diversity of experiences declines with age. We further provide methodological (e.g., quantification, measurement, timescales), theoretical (e.g., moderators, cross-domain considerations, contexts of ontogenetic and sociohistorical change processes) considerations for future studies of experiential diversity beyond the daily domains. X-versity theory provides a theoretical leap in lifespan socioemotional aging, taking advantage of rich categorical data measured in many studies of daily life, and allowing for more nuanced consideration of the role of diverse experiences for healthy aging.

